# Genetic Modification of Human Peripheral Blood Aspirates Using Recombinant Adeno‐Associated Viral Vectors for Articular Cartilage Repair with a Focus on Chondrogenic Transforming Growth Factor‐β Gene Delivery

**DOI:** 10.5966/sctm.2016-0149

**Published:** 2016-08-29

**Authors:** Janina Frisch, Patrick Orth, Jagadeesh Kumar Venkatesan, Ana Rey‐Rico, Gertrud Schmitt, Dieter Kohn, Henning Madry, Magali Cucchiarini

**Affiliations:** ^1^ Center of Experimental Orthopaedics, Saarland University Medical Center, Homburg/Saar, Germany; ^2^ Department of Orthopaedic Surgery, Saarland University Medical Center, Homburg/Saar, Germany

**Keywords:** Cartilage repair, Gene therapy, Peripheral blood aspirates, rAAV vectors, Transforming growth factor‐β

## Abstract

Transplantation of genetically modified peripheral blood aspirates that carry chondrogenically competent progenitor cells may offer new, convenient tools to treat articular cartilage lesions compared with the more complex and invasive application of bone marrow concentrates or of bone marrow‐derived mesenchymal stem cells. Here, we show that recombinant adeno‐associated viral (rAAV) vectors are powerful gene vehicles capable of successfully targeting primary human peripheral blood aspirates in a stable and safe manner, allowing for an efficient and long‐term transgene expression in such samples (up to 63 days with use of a *lacZ* reporter gene and for at least 21 days with application of the pleiotropic, chondrogenic factor transforming growth factor‐β [TGF‐β]). rAAV‐mediated overexpression of TGF‐β enhanced both the proliferative and metabolic properties of the peripheral blood aspirates, also increasing the chondrogenic differentiation processes in these samples. Hypertrophy and osteogenic differentiation events were also activated by production of TGF‐β via rAAV, suggesting that translation of the current approach in vivo will probably require close regulation of expression of this candidate gene. However, these results support the concept of directly modifying peripheral blood as a novel approach to conveniently treat articular cartilage lesions in patients. Stem Cells Translational Medicine *2017;6:249–260*

**Significance Statement**

The present study evaluated a novel, clinically relevant approach based on the use of genetically modified peripheral blood aspirates as a potent, less invasive source of chondroreparative progenitor cells (compared with isolated mesenchymal stem cells or bone marrow aspirates) to further improve the healing of cartilage injuries in patients. The results demonstrate that gene transfer in human peripheral blood aspirates can be successfully performed by using clinically adapted recombinant adeno‐associated viral vectors, leading to the stimulation of chondrogenic events upon overexpression of the chondrogenic TGF‐β. This approach forms a promising basis for the further development of novel therapeutic options to treat articular cartilage defects by transplantation of genetically modified blood in vivo upon controlled regulation of gene expression.

## Introduction

The articular cartilage is the tissue that promotes the smooth gliding of the articulating surfaces in diarthrodial joints 1. The lack of vascularization in adult cartilage, and thus of chondroreparative/chondroregenerative cells, such as mesenchymal stem cells (MSCs), which may contribute to the natural healing responses to injury, is a critical issue for the reproduction of a natural, hyaline cartilage in sites of cartilage damage 2. Lesions to the cartilage are diverse and include focal (chondral and osteochondral) defects resulting from trauma and generalized osteoarthritis (OA) (a progressive disease with an irreversible deterioration of the cartilage but also of other joint tissues, such as bone, synovium, meniscus, tendons/ligaments, and muscles). Current surgical options to treat focal lesions include marrow stimulation to provide access to regenerative cells from the subchondral bone in the lesions (subchondral drilling, microfracture, abrasion arthroplasty) and autologous chondrocyte implantation (ACI) with or without supportive matrices for chondral defects and transplantation of osteochondral cylinders or of subchondral bone grafts with ACI for deep, osteochondral defects, whereas OA is managed by osteotomies or joint replacement 3. Yet, although new tissue is formed in the lesions with such procedures, its fibrocartilaginous nature (it consists of type I collagen vs. the type II collagen and proteoglycans typical of the hyaline cartilage) does not allow for durable mechanical competence or for integrity of the repair tissue with the adjacent cartilage 3. Thus, improved treatments are needed.

Interestingly, several lines of evidence recently showed the presence of chondrogenically competent MSCs in the peripheral blood compartment of humans (PB‐MSCs) 4, 5 as an easily accessible source of cells capable of promoting cartilage repair 6, 7, 8, 9. Although these trials had promising results, reconstitution of the original hyaline cartilage was still not fully achieved, possibly because of the low representation of MSCs in the blood (0.0002%) 10. An attractive approach to enhance the potency of such PB‐MSCs would be to modify them by gene transfer methods upon delivery of chondroreparative sequences and using conditions of maintenance in a natural cellular and biological microenvironment (i.e., peripheral blood aspirates containing biochemical factors and other cell types, such as hematopoietic cells and fibroblasts, having crosstalks with PB‐MSCs) 11. In this regard, administration of recombinant adeno‐associated viral (rAAV) vectors might be best suited because these constructs are clinically adapted, promoting high and persistent levels of transgene expression in various human musculoskeletal cell populations 12, 13, 14, 15, 16, 17, 18 compared with other less efficient or more immunogenic/toxic vectors (nonviral, adenoviral, retroviral/lentiviral vectors) 19, 20, 21, 22, 23, 24, 25, 26. Although chondrogenic gene transfer has been successfully performed via rAAV in aspirates from the bone marrow of patients by using transforming growth factor‐β (TGF‐β) 27, insulin‐like growth factor‐I (IGF‐I) 28, and the cartilage‐specific SOX9 transcription factor 29, no evidence thus far demonstrates the possibility of modifying human peripheral blood aspirates with this vector class. The goal of the present study was therefore to evaluate the ability of rAAV to directly transduce human peripheral blood aspirates, with a focus on delivering a TGF‐β gene as a means to stimulate the chondrogenic differentiation processes in such samples compared with control treatments.

For the first time, to our best knowledge, we report that peripheral blood aspirates can be efficiently, stably, and safely modified by rAAV‐mediated gene transfer, allowing overexpression of a candidate TGF‐β sequence capable of stimulating the proliferative, metabolic, and chondrogenic differentiation processes in such samples. Because hypertrophy and osteogenic differentiation events were also triggered in the aspirates, tight control of TGF‐β transgene expression will most likely be necessary upon application of the current therapeutic construct in vivo. Nevertheless, these findings show the value of genetically modifying peripheral blood aspirates for future translational applications to treat cartilage lesions in patients.

## Materials and Methods

### Reagents

All reagents were from Sigma‐Aldrich (Munich, Germany, http://www.sigmaaldrich.com), unless otherwise indicated. The recombinant TGF‐β was from Peprotech (Hamburg, Germany, https://www.peprotech.com). Dimethylmethylene blue (DMMB) was purchased at Serva (Heidelberg, Germany, http://www.serva.de). The anti‐β‐gal (GAL‐13) and anti‐type X collagen (COL‐10) antibodies were from Sigma‐Aldrich; the anti‐TGF‐β (V), anti‐SOX9 (C‐20), anti‐CD105 (T‐20), and anti‐CD34 (C‐18) antibodies were from Santa Cruz Biotechnology (Heidelberg, Germany, http://www.scbt.com); the anti‐type II collagen (II‐II6B3) antibody was from the National Institutes of Health Hybridoma Bank (University of Iowa, Ames, IA, http://www.dshb.biology.uiowa.edu); and the anti‐type I collagen (AF‐5610) antibody was from Acris Antibodies (Hiddenhausen, Germany, https://www.acris‐antibodies.com/). The human TGF‐β (hTGF‐β Quantikine enzyme‐linked immunosorbent assay (ELISA) was purchased at R&D Systems (Wiesbaden, Germany, https://www.rndsystems.com/). The Cytotoxicity Detection Kit (lactate dehydrogenase [LDH]) was obtained from Roche Applied Science (Mannheim, Germany, http://www.roche‐applied‐science.com). The type II‐, I, and X collagen ELISAs were from Antibodies‐Online (Aachen, Germany, http://www.antibodies‐online.com).

### Peripheral Blood Aspirates

Peripheral blood (∼3 ml) was aspirated during routine blood collection in donors undergoing total knee arthroplasty (*n* = 4; mean age ± SD, 50 ± 17 years) with informed consent. All procedures were in accordance with the Helsinki Declaration. The study was approved by the Ethics Committee of the Saarland Physicians Council (application 39/14).

### Plasmids and rAAV Vectors

An AAV‐2 genomic clone (pSSV9) 30, 31 served as the template for all constructs used in the study. rAAV‐*lacZ* carries the *lacZ* gene encoding the *Escherichia coli* β‐galactosidase (β‐gal) and rAAV‐hTGF‐β, an hTGF‐β cDNA (1.2 kb), both under the control of the cytomegalovirus immediate‐early promoter 27, 32. Recombinant vectors (rAAV) were packaged as conventional (not self‐complementary) vectors by using the 293 adenovirus‐transformed embryonic kidney cell line and helper functions provided by Adenovirus 5 and pAd8 helper plasmid as previously described 17. Vector preparations were purified, dialyzed, and titrated via real‐time polymerase chain reaction (PCR), resulting in 10^10^ transgene copies/ml with approximately 1/500 functional recombinant viral particles 17, 27, 32.

### rAAV‐Mediated Gene Transfer

Immediately after collection, the peripheral blood aspirates were divided into aliquots (100 μl/well in 96‐well plates) and transduced with 40‐μl vectors (8 × 10^5^ functional recombinant viral particles, multiplicity of infection [MOI], 10 ± 3), followed by an addition of 50 μl of supplement‐free DMEM 27. The aspirates were incubated for 90 minutes at 37°C and 5% CO_2_, with subsequent addition of 60 μl of DMEM, 10% fetal bovine serum (growth medium), or chondrogenic medium to promote chondrogenesis (4.5 g/L DMEM high glucose, 100 U/ml penicillin, 100 μl/ml streptomycin, 6.25 μg/ml insulin, 6.25 μg/ml transferrin, 6.25 μg/ml selenous acid, 5.35 μg/ml linoleic acid, 1.25 μg/ml bovine serum albumin, 1 mM sodium pyruvate, 37.5 μg/ml ascorbate 2‐phosphate, 10^−7^ M dexamethasone, and 10 ng/ml TGF‐β3). Incubation was pursued for up to 21 days, with careful medium change once per week 27.

### Transgene Expression

Detection of *lacZ* expression was performed by X‐Gal staining (Roche Applied Science) and by immunohistochemistry using a specific primary antibody, a biotinylated secondary antibody, and diaminobenzidine (DAB) as the chromogen via the ABC method (Vector Laboratories, Alexis Deutschland GmbH, Grünberg, Germany, https://vectorlabs.com) 27, 29. The production of TGF‐β was monitored by ELISA and via immunohistochemistry. A condition with omission of the primary antibody was included as a control to check for secondary immunoglobulins. Sections were examined under light microscopy (Olympus BX45, Olympus, Hamburg, Germany, https://www.olympus‐europa.com). Absorbance was evaluated by using a GENios spectrophotometer (Tecan, Crailsheim, Germany, http://www.tecan.com/).

### Cytotoxicity Assay

Cytotoxic events were assessed by using the Cytotoxicity Detection Kit (LDH) by measuring the release of LDH activity in the aspirates according to the manufacturer's instructions and as previously described 29. Absorbance was monitored on a GENios spectrophotometer (Tecan). The data were calculated as percentage cytotoxicity indices, calculated by using the following formula:

(1) [experimental value‐low control]/[high control‐low control]×100

In this formula, “low control” corresponds to cells without vector treatment and “high control” to cells placed in lysis buffer provided in the kit 29.

### Biochemical Analyses

Aspirates were collected after 21 days in a total volume of 100 μl of fresh, supplement‐free DMEM and digested with papain (final concentration, 75 μg/ml) at 60°C for ≥60 minutes 27. The DNA contents were measured by Hoechst 22358 assay; the proteoglycan contents were determined by binding to DMMB; and the type II, I, and X collagen contents determined by specific ELISAs 27. A protein assay (Pierce, ThermoFisher Scientific Life Sciences, Schwerte, Germany, http://www.thermofisher.com) was used to monitor total cellular proteins for normalization. All measurements were performed on a GENios spectrophotometer/fluorometer (Tecan).

### Histological and Immunohistochemical Analyses

Aspirates were collected after 21 days, fixed in 4% formalin, dehydrated in graded alcohols, embedded in paraffin, and sectioned at 3 μm. Histological staining was performed to monitor cell structure and densities (hematoxylin and eosin [H&E]), matrix proteoglycans (toluidine blue), and matrix mineralization (alizarin red) 27. Expression of type II, I, and X collagen and of SOX9 was determined by immunohistochemistry using specific primary antibodies, biotinylated secondary antibodies, and the ABC method with DAB as the chromogen 27. Control conditions were included by omitting the primary antibodies. Sections were examined under light microscopy (Olympus BX45). Immunophenotyping analyses were performed by using fluorescent secondary antibodies (Fluorescein FI2000, Texas Red TI5000) from Vector Laboratories (Burlingame, CA).

### Histomorphometry

To monitor the cell densities, the total cell numbers per standardized area (cells per mm^2^) and total pixels per image were determined on H&E‐stained histological sections 27. The toluidine blue and alizarin red staining intensities and those for type II, I, and X collagen and SOX9 immunostaining were monitored at magnification of ×20 by inverting the pictures to grayscale mode, adapting background DAB signal for comparable range, and measuring the mean gray value per total area covered with cells (pixels per mm^2^) 27. All data were collected at three random standardized sites or using 10 serial histological and immunohistochemical sections for each parameter, test, and replicate condition using the cellSens Standard program (Olympus) and Adobe Photoshop (Adobe Systems, Unterschleissheim, Germany, http://www.adobe.com).

### Real‐Time Reverse Transcriptase PCR Analyses

Total cellular RNA was extracted from peripheral blood aspirates in a total volume of 100 μl of fresh, supplement‐free DMEM at day 21 after transduction by using the TRIzol reagent (ThermoFisher Scientific Life Sciences, Darmstadt, Germany) and RNeasy Protect Mini Kit (Qiagen, Hilden, Germany, https://www.qiagen.com). An on‐column RNase‐free DNase treatment (Qiagen) was included in the procedure, and final RNA elution was performed in 30 μl of RNase‐free water. Reverse transcription was next carried out with 8 μl of extracted RNA using the first‐strand cDNA synthesis kit for reverse transcriptase (RT)‐PCR avian myeloblastosis virus (AMV) (Roche Applied Science).

The resulting cDNA products (2 μl per sample and condition) were finally amplified by real‐time RT‐PCR with Brilliant SYBR Green QPCR Master Mix (Stratagene, Agilent Technologies, Waldbronn, Germany, http://www.agilent.com) on an Mx3000P QPCR operator system (Stratagene) under the following conditions: 95°C for 10 minutes, amplification by 55 cycles (denaturation at 95°C, 30 seconds; annealing at 55°C, 1 minute; extension at 72°C, 30 seconds), denaturation (95°C, 1 minute), and final incubation (55°C, 30 seconds). Primers (ThermoFisher Scientific Life Sciences, Darmstadt, Germany) for selected gene profiles were applied at a final concentration of 150 nm as follows: aggrecan (ACAN) (chondrogenic marker) (forward 5′‐GAGATGGAGGGTGAGGTC‐3′; reverse 5′‐ACGCTGCCTCGGGCTTC‐3′), type II collagen (COL2A1) (chondrogenic marker) (forward 5′‐GGACTTTTCTCCCCTCTCT‐3′; reverse 5′‐GACCCGAAGGTC TTACAGGA‐3′), SOX9 (chondrogenic marker) (forward 5′‐ACACACAGCT CACTCGACCTTG‐3′; reverse 5′‐GGGAATTCTGGTTGGTCCTCT‐3′), type I collagen (COL1A1) (osteogenic marker) (forward 5′‐ACGTCCTGGTGAAGTTGGTC‐3′; reverse 5′‐ACCAGGGAAGCCTCTCTCTC‐3′), type X collagen (COL10A1) (marker of hypertrophy) (forward 5′‐CCCTCTTGTTAGTGCCAACC‐3′; reverse 5′‐AGATTCCAGTCCTTGGGTCA‐3′), matrix metalloproteinase 13 (MMP13) (marker of terminal differentiation) (forward 5′‐AATTTTCACTTTTGGCAATGA‐3′; reverse 5′‐CAAATAATTTATGAAAAAGGGATGC‐3′), alkaline phosphatase (ALP) (osteogenic marker) (forward 5′‐TGGAGCTTCAGAAGCTCAACACCA‐3′; reverse 5′‐ATCTCGTTGTCTGAG TACCAGTCC‐3′), runt‐related transcription factor 2 (RUNX2) (osteogenic marker) (forward 5′‐GCAGTTCCCAAGCATTTCAT‐3′; reverse 5′‐CACTCTGGCTTTGGGA AGAG‐3′), and glyceraldehyde‐3‐phosphate dehydrogenase (GAPDH) (housekeeping gene and internal control) (forward 5′‐GAAGGTGAAGGTCGGAGTC‐3′; reverse 5′‐GAAGATGGTGATGGGATTTC‐3′) 27. Water and nonreverse transcribed mRNA were included as controls. Threshold cycle (Ct) values were measured on the MxPro QPCR software (Stratagene), and all values were normalized to GAPDH expression by using the 2^−ΔΔCt^ method 27.

### Statistical Analysis

Each condition was performed (SPSS version 23; IBM, Armonk, NY, USA, http://www.ibm.com/us‐en/) in duplicate in two independent experiments for each patient. Data are expressed as mean ± SD of separate experiments. The *t* test was used where appropriate. Any *p* value <.05 was considered to indicate a statistically significant difference.

## Results

### Effective and Safe Genetic Modification of Human Peripheral Blood Aspirates via rAAV Vectors

**Figure 1:**
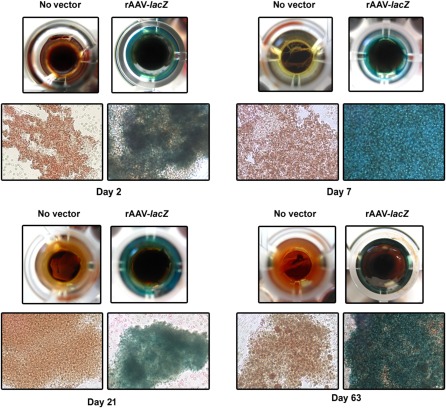
Detection of transgene expression in human peripheral blood aspirates after rAAV‐mediated gene transfer. Aspirates were transduced with rAAV‐*lacZ* or rAAV‐human transforming growth factor‐β (40 μl each vector) or left untreated, kept in regular growth medium, and processed to monitor transgene (*lacZ*) expression by X‐Gal staining at the denoted time points (original magnification, ×40; all representative data) as described in Materials and Methods. Abbreviation: rAAV, recombinant adeno‐associated viral.

Human peripheral blood aspirates were first transduced with a reporter gene vector (rAAV‐*lacZ*) in regular growth medium culture conditions to determine the ability of this vector class to modify such samples during an extended period compared with control (untreated) condition. Robust, homogeneous, and stable *lacZ* expression was observed in the aspirates upon *lacZ* treatment 2 days after transduction and for at least 63 days, the longest time point evaluated, whereas expression was never noted in the absence of reporter vector (Fig. 1).

We next examined whether administration of rAAV led to cytotoxic events in the aspirates. We focused on a 21‐day period of evaluation reflecting the standard phase of chondrogenic differentiation 27. Mean cytotoxicity indices never exceeded 6% in the aspirates treated (*lacZ*, TGF‐β) or not treated with rAAV at any time point of the analysis, without a significant deleterious effect of the vectors relative to the condition in which the vectors were omitted (*p* ≥ .054).

### Genetic Modification of Human Peripheral Blood Aspirates by rAAV‐Mediated Gene Transfer in Conditions of Chondrogenic Induction

Human peripheral blood aspirates were next transduced with the reporter (*lacZ*) and candidate (TGF‐β) rAAV vectors to further evaluate the feasibility of modifying such samples with this vector type in conditions of chondrogenic induction during a standard period of chondrogenic evaluation (21 days) 27 and relative to control condition (absence of vector treatment).

**Figure 2:**
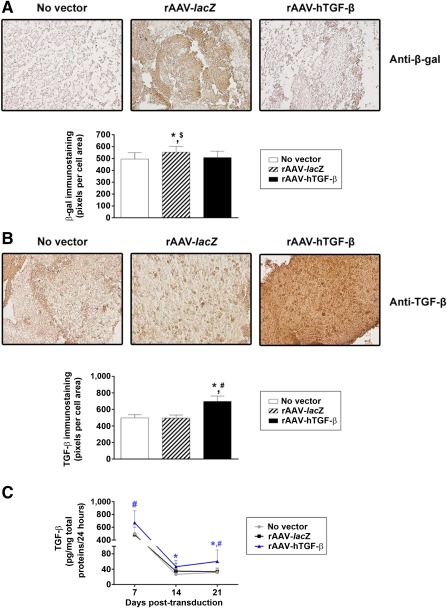
Detection of transgene expression in chondrogenically induced human peripheral blood aspirates after rAAV‐mediated gene transfer. Aspirates were transduced with rAAV‐*lacZ* or rAAV‐hTGF‐β or left untreated (as described in Fig. 1), kept in chondrogenic medium for 21 days, and processed to monitor **(A)** *lacZ* expression by immunohistochemistry with corresponding histomorphometric analyses and **(B, C)** transforming growth factor‐β expression by immunohistochemistry with corresponding histomorphometric analyses **(B)** and enzyme‐linked immunosorbent assay **(C)** as described in Materials and Methods (original magnification, ×20; all representative data). ∗, statistically significant compared with no vector treatment; #, significant compared with rAAV‐*lacZ*; $, significant compared with rAAV‐hTGF‐β. Abbreviations: hTGF‐β, human transforming growth factor‐β; rAAV, recombinant adeno‐associated viral; TGF‐β, transforming growth factor‐β.

Strong, specific *lacZ* expression was noted in the aspirates when rAAV‐*lacZ* was applied compared with the other conditions (rAAV‐hTGF‐β, no vector) (Fig. 2A), a finding corroborated by measurements of the intensity of immunostaining (up to 1.1‐fold difference; *p* ≤ .001) (Fig. 2A). TGF‐β was also overexpressed over time in the samples transduced with rAAV‐hTGF‐β, as observed by the intense immunohistochemical signal specific to the growth factor on sections from aspirates compared with control (rAAV‐*lacZ*, no vector) treatments (Fig. 2B). This finding was corroborated by the results of a histomorphometric analysis (up to a 1.4‐fold difference; *p* ≤ .001) (Fig. 2B) and a specific TGF‐β ELISA (up to a 1.9‐ fold difference; *p* ≤ .001) (Fig. 2C).

### Overexpression of TGF‐β via rAAV Enhanced the Proliferative, Metabolic, and Chondrogenic Differentiation Activities in Chondrogenically Induced Human Peripheral Blood Aspirates

We then examined the potential effects of the candidate rAAV TGF‐β treatment on the biological and differentiation processes in human peripheral blood aspirates after 21‐day chondrogenic induction relative to control (*lacZ*, no vector treatment) conditions.

**Figure 3:**
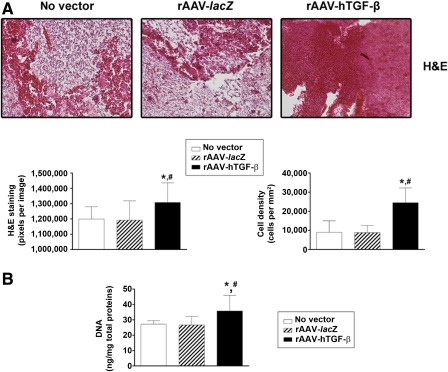
Evaluation of the proliferative activities in chondrogenically induced human peripheral blood aspirates upon rAAV‐mediated gene transfer. Aspirates were transduced with rAAV‐*lacZ* or rAAV‐hTGF‐β or left untreated (as described in Figs. 1 and 2), kept in chondrogenic medium for 21 days, and processed to evaluate **(A)** the cell densities on H&E‐stained histological sections with corresponding histomorphometric analyses (original magnification, ×20; representative data) and **(B)** the DNA contents as described in Materials and Methods. ∗, statistically significant compared with no vector treatment; #, significant compared with rAAV‐*lacZ*. Abbreviations: H&E, hematoxylin and eosin; hTGF‐β, human transforming growth factor‐β; rAAV, recombinant adeno‐associated viral; TGF‐β, transforming growth factor‐β.

**Figure 4:**
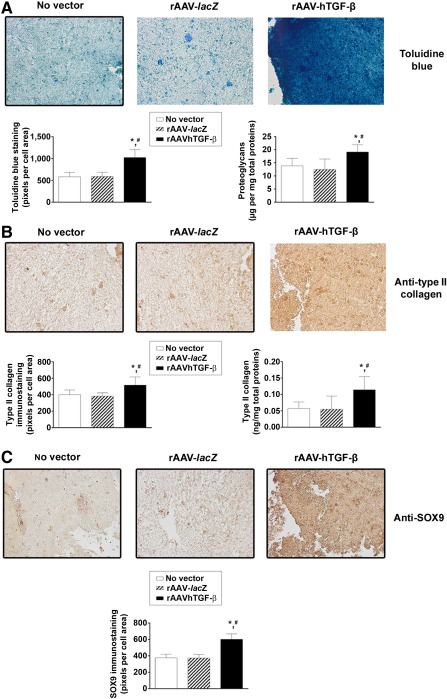
Determination of the chondrogenic processes in chondrogenically induced human peripheral blood aspirates upon rAAV‐mediated gene transfer. Aspirates were transduced with rAAV‐*lacZ* or rAAV‐hTGF‐β or left untreated (as described in Figs. 1–3), kept in chondrogenic medium for 21 days, and processed for **(A)** toluidine blue staining with corresponding histomorphometric analyses and measurement of the proteoglycan contents, **(B)** type II collagen immunostaining with corresponding histomorphometric analyses and measurement of the type II collagen contents, and **(C)** SOX9 immunostaining with corresponding histomorphometric analyses, as described in Materials and Methods (original magnification, ×20; all representative data). ∗, Statistically significant compared with no vector treatment; #, significant compared with rAAV‐*lacZ*. Abbreviations: hTGF‐β, human transforming growth factor‐β; rAAV, recombinant adeno‐associated viral.

Administration of rAAV‐hTGF‐β led to significantly higher cell densities in the aspirates over time compared with the controls, as noted on H&E‐stained and histomorphometrically processed sections of aspirates (up to a 2.8‐fold difference; *p* ≤ .020) (Fig. 3). Similar results were obtained when we monitored the DNA contents in the aspirates (up to a 1.3‐fold difference with TGF‐β; *p* ≤ .042) (Fig. 3). In addition, treatment with rAAV TGF‐β significantly enhanced the deposition of cartilage‐specific components and markers, with more robust toluidine blue staining (Fig. 4A) and type II collagen and SOX9 immunostaining (Fig. 4B, 4C, respectively) relative to the control conditions. Again, these results were substantiated by a histomorphometric estimation of the intensities of staining and immunostaining (up to 1.8‐, 1.3‐, and 1.6‐fold differences for toluidine blue, type II collagen, and SOX9, respectively; *p* ≤ .001) (Fig. 4A–4C). In good agreement, the proteoglycan and type II collagen contents were higher with TGF‐β than with *lacZ* and the no‐vector treatment (up to 1.5‐ and 2.1‐fold differences for the proteoglycans and type II collagen, respectively; *p* ≤ .028) (Fig. 4A, 4B).

### Effects of TGF‐β Gene Transfer via rAAV on the Hypertrophic and Osteogenic Differentiation Processes in Chondrogenically Induced Human Peripheral Blood Aspirates

Chondrogenically induced aspirates were also analyzed for a potential influence of the gene transfer and overexpression of TGF‐β with rAAV on the development of hypertrophy and osteogenic differentiation relative to control (*lacZ*, absence of vector) treatments.

**Figure 5:**
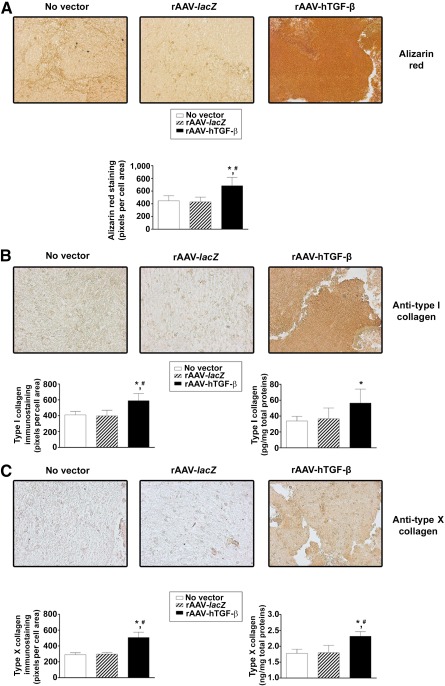
Evaluation of hypertrophic and terminal differentiation events in chondrogenically induced human peripheral blood aspirates upon rAAV‐mediated gene transfer. Aspirates were transduced with rAAV‐*lacZ* or rAAV‐hTGF‐β or left untreated (as described in Figs. 1–4), kept in chondrogenic medium for 21 days, and processed for **(A)** alizarin red staining with corresponding histomorphometric analyses, **(B)** type I collagen immunostaining with corresponding histomorphometric analyses and measurement of the type I collagen contents, and **(C)** type X collagen immunostaining with corresponding histomorphometric analyses and measurement of the type X collagen contents, as described in Materials and Methods (original magnification, ×20; all representative data). ∗, Statistically significant compared with no vector treatment; #, significant compared with rAAV‐*lacZ*. Abbreviations: hTGF‐β, human transforming growth factor‐β; rAAV, recombinant adeno‐associated viral.

Of note, application of rAAV‐hTGF‐β enhanced the deposition of hypertrophic and osteogenic components and markers over time, with stronger alizarin red staining (Fig. 5A) and type I and type X collagen immunostaining (Fig. 5B, 5C, respectively) relative to the control conditions. Concordant with that, a histomorphometric analysis demonstrated higher staining and immunostaining intensities for alizarin red, type I and type X collagen (up to 1.6‐, 1.5‐, and 1.7‐fold differences, respectively; *p* ≤ .001) (Fig. 5A–5C). These results were further supported by an evaluation of the type I and type X collagen contents, revealing significantly higher production levels for these components upon TGF‐β gene transfer versus *lacZ* and the no‐vector treatment (up to 1.7‐ and 1.3‐fold differences for type I and type X collagen, respectively; *p* ≤ .025 except for type I collagen comparing TGF‐β vs. *lacZ* transduction, for which *p* = .062) (Fig. 5B, 5C).

### Gene Expression Profiles in Chondrogenically Induced Human Peripheral Blood Aspirates After rAAV Gene Transfer

Chondrogenically induced aspirates were analyzed for the expression of cartilage‐, hypertrophic‐, and osteogenic‐specific markers over time (21 days) by real‐time RT‐PCR to strengthen the findings reported earlier in the text.

**Figure 6:**
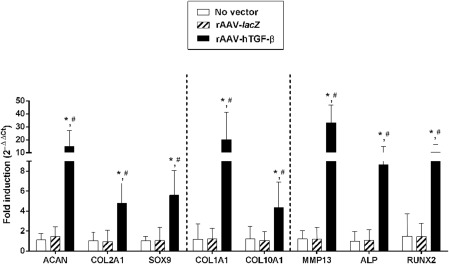
Real‐time reverse transcriptase polymerase chain reaction analyses in chondrogenically induced human peripheral blood aspirates upon rAAV‐mediated gene transfer. Aspirates were transduced with rAAV‐*lacZ* or rAAV‐hTGF‐β or left untreated (as described in Figs. 1–5), kept in chondrogenic medium for 21 days, and analyzed for marker gene expression of ACAN, COL2A1, the transcription factor SOX9, COL1A1, COL10A1, MMP13, ALP, and the transcription factor RUNX2, with glyceraldehyde‐3‐phosphate dehydrogenase serving as a housekeeping gene and internal control for normalization. Ct values were generated for each target gene, and fold inductions (relative to untreated aspirates) were measured by using the 2^−ΔΔCt^ method, as described in Materials and Methods. ∗, Statistically significant compared with no vector treatment; #, significant compared with rAAV‐*lacZ*. Abbreviations: ACAN, aggrecan; ALP, alkaline phosphatase; COL1A1, type I collagen; COL2A1, type II collagen; hTGF‐β, human transforming growth factor‐β; MMP13, matrix metalloproteinase 13; rAAV, recombinant adeno‐associated viral; RUNX2, runt‐related transcription factor 2.

Importantly, application of the reporter rAAV‐*lacZ* vector had no detrimental influence on the chondrogenic gene expression profiles of the aspirates compared with the control (untreated) condition (*p* ≥ .277) (Fig. 6). In contrast, overexpression of TGF‐β led to higher expression levels of such markers relative to control treatments over time (up to 13.5‐, 4.6‐, and 5.3‐fold differences for ACAN, COL2A1, and SOX9, respectively; *p* ≤ .016) (Fig. 6), in good agreement with the results of the histomorphometric and biochemical evaluations.

Treatment with *lacZ* also did not alter the hypertrophic and osteogenic gene expression profiles compared with the condition in which the vector was omitted (*p* ≥ .374) (Fig. 6). Again concordant with the histomorphometric and biochemical findings, administration of rAAV‐hTGF‐β enhanced the expression levels of such markers versus control treatments over time (17‐, 3.5‐, and 8.6‐fold differences for COL1A1, COL10A1, and ALP, respectively; *p* ≤ .041) (Fig. 6). Interestingly, these effects were accompanied by increases in the expression of MMP13 (marker of terminal differentiation) (27‐fold difference; *p* ≤ .001) and of RUNX2 (transcription factor controlling the osteoblastic expression of COL1, COL10, and MMP13) (6.9‐fold difference; *p* ≤ .008) (Fig. 6).

### Immunophenotypic Analyses in Chondrogenically Induced Human Peripheral Blood Aspirates After rAAV Gene Transfer

**Figure 7:**
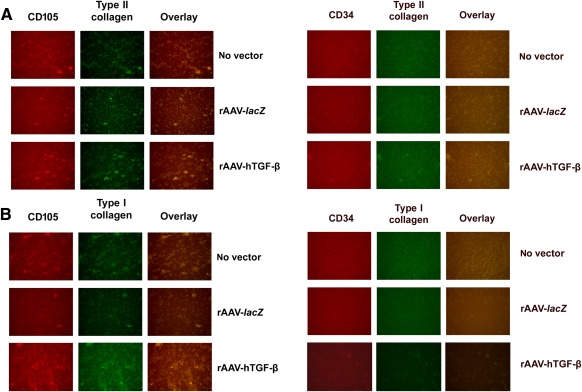
Immunophenotypic analyses in chondrogenically induced human peripheral blood aspirates upon rAAV‐mediated gene transfer. Aspirates were transduced with rAAV‐*lacZ* or rAAV‐hTGF‐β or left untreated (as described in Figs. 1–6), kept in chondrogenic medium for 21 days, and analyzed for the coexpression of **(A)** CD105/type II collagen versus CD34/type II collagen and **(B)** CD105/type I collagen versus CD34/type I collagen by specific coimmunostaining, as described in Materials and Methods (original magnification, ×20; all representative data). Abbreviations: hTGF‐β, human transforming growth factor‐β; rAAV, recombinant adeno‐associated viral.

Chondrogenically induced aspirates were finally processed to analyze the coexpression of CD105/type II or type I collagen versus CD34/type II or type I collagen to determine the cell population undergoing specific lineage commitment in such samples over time (21 days) (Fig. 7).

Remarkably, expression of chondrogenic (type II collagen) (Fig. 7A) and osteogenic (type I collagen) (Fig. 7B) markers was restricted to CD105^+^ cells, whereas no expression was noted in CD34^+^ cells. Of further note, higher levels of type II and type I collagen expression were observed upon administration of rAAV‐hTGF‐β relative to control treatments (rAAV‐*lacZ*, no vector condition), strongly corroborating the findings of the histological, immunohistochemical, biochemical, and real‐time RT‐PCR evaluations.

## Discussion

Gene transfer to enhance the chondrogenic potency of peripheral blood aspirates represents a promising new area of translational research that may provide single‐step, convenient approaches to treat cartilage defects upon implantation within sites of lesions 11. In the present study, we investigated the feasibility of targeting human peripheral blood aspirates by rAAV vectors as a means to enhance the chondrogenic responses to therapeutic TGF‐β treatment 33, 34, 35.

The data first indicate that rAAV vectors can effectively promote high, sustained levels of transgene expression in primary human peripheral blood aspirates (up to 63 days for *lacZ* and at least 21 days for TGF‐β), concordant with findings in human bone marrow aspirates when applying the same vector construct 27, 29. Interestingly, while similar amounts of TGF‐β were produced early on in these different samples at similar vector doses (MOI, 10 ± 3) (∼671 and 511 pg rhTGF‐β/mg total proteins/24 hours on day 7 in aspirates from the peripheral blood and bone marrow, respectively; *p* = 1.000) 27, the concentrations of growth factor became higher in the bone marrow aspirates at an intermediate stage of induction (∼47 vs. 113 pg rhTGF‐β/mg total proteins/24 hours on day 14 in aspirates from the peripheral blood and bone marrow, respectively [i.e., a 2.4‐fold difference]; *p* ≤ .001) but lower at the latest stage of evaluation (∼60 vs. 16 pg rhTGF‐β/mg total proteins/24 hours on day 21 in aspirates from the peripheral blood and bone marrow, respectively [i.e., a 3.8‐fold difference]; *p* ≤ .001).

Such discrepancies may reflect differences in the representation of chondrogenically competent MSCs in the samples (0.0002% in the peripheral blood vs. ∼1% in the bone marrow) 10 that may principally undergo differentiation under continuous chondrogenic stimulation and may thus be the principal source of growth factor over time 27, 28, dissimilar cellular and biological microenvironments in the aspirates that may affect maintenance and differentiation 4, 10, 36, 37, 38, variable amounts of cell surface receptor (a heparin sulfate proteoglycan) 39 and cell‐specific coreceptors to rAAV transduction 40, 41, 42, and/or disparities in rAAV transgene intracellular processing and expression 43, 44. Work is ongoing to identify and characterize the subpopulations subjected to gene transfer and committing toward the chondrocyte phenotype over time in the peripheral blood aspirates to address such hypotheses.

We next found that TGF‐β overexpression via rAAV was capable of stimulating the proliferative and chondrogenic processes in peripheral blood aspirates, again in good agreement with previous findings when the current construct was applied to human bone marrow aspirates 27. Of further interest, the indices of proliferation in the peripheral blood aspirates were lower than those noted in the bone marrow aspirates after TGF‐β treatment (∼36 ng DNA/mg total proteins vs. 3.6 μg DNA/mg total proteins on day 21, respectively [i.e., a 100‐fold difference]; *p* ≤ .001), with a magnitude of effect again more significant in the bone marrow aspirates when TGF‐β was compared to control treatment (1.3‐ vs. 5.1‐fold increased DNA contents with TGF‐β relative to *lacZ* in aspirates from the peripheral blood and bone marrow, respectively [i.e., a 3.9‐fold difference]; *p* ≤ .001) 27. This might be again due to the lower representation of chondrogenically competent MSCs in the peripheral blood, to distinct biological microenvironments (presence or absence of other mitogenic factors in the blood vs. the bone marrow) and/or to a possible (positive/negative) influence of other cell populations in the aspirates that might be specific to the bone marrow compared with the peripheral blood (paracrine/autocrine effects). Strikingly, although the proteoglycans contents were higher in peripheral blood aspirates compared with those from the bone marrow upon TGF‐β treatment (∼19 μg/mg total proteins vs. 64 ng/mg total proteins on day 21, respectively [i.e., a 297‐fold difference]; *p* ≤ .001), those for type II collagen were lower (∼0.120 vs. 3 ng/mg total proteins in aspirates from the peripheral blood and bone marrow on day 21, respectively [i.e., a 25 × 10^3^‐fold difference]; *p* ≤ .001) at similar magnitudes compared with each respective control treatment (∼2‐fold difference) 27.

Such discrepancies might again result from the distinct repartition of chondrogenically competent MSCs in the aspirates and/or from their different state of activation upon chondrogenic expansion, from specific biological balance (anabolism/catabolism, inflammation) and cellular interplays in the blood versus bone marrow, and/or from the timing of full matrix deposition, as noted in the bone marrow in late (between 40 and 50 days) versus earlier (21 days) time points 45, 46. Work is again ongoing here to test these hypotheses by evaluating a possible influence of the biochemical environment in the aspirates and in their cell subpopulations over extended periods of chondrogenic stimulation. Of note, we also showed that expression of chondrogenic markers was typical of CD105^+^ cells, supporting the concept that mostly MSCs competently commit toward the chondrogenic phenotype in the marrow under prolonged, continuous lineage‐specific stimulation.

Finally, we demonstrated that rAAV‐mediated TGF‐β gene transfer and overexpression enhanced the levels of hypertrophic differentiation in the peripheral blood aspirates, probably because of enhanced gene expression profiles of MMP13 (marker of terminal differentiation), ALP (osteogenic marker), and RUNX2 (transcription factor controlling the osteoblastic expression of COL1, COL10, and MMP13). This is in contrast with the effects noted when the same construct was applied to bone marrow aspirates, wherein TGF‐β instead delayed hypertrophic processes 27, possibly because of a different timing of differentiation and/or again a probably diverse cell composition and environment in the aspirates with detrimental/beneficial factors, distinct pathways of induction 47, and cellular crosstalks. However, little is still known about the mechanisms of action of the components and interplays between the various cell subpopulations in these different samples. In addition, osteogenic differentiation was specific for the CD105^+^ (MSCs) subpopulation, suggesting again that mostly MSCs undergo this type of commitment in the marrow under prolonged, continuous chondrogenic stimulation.

The present study provides evidence of the potential of rAAV vectors to genetically modify human peripheral blood aspirates as a means to enhance chondrogenic differentiation processes effectively and over time especially by overexpressing TGF‐β for future convenient transplantation in clinically relevant, orthotopic sites of cartilage defect 10, 48, 49. Such a translational approach will require the precise regulation of TGF‐β production to prevent undesirable hypertrophic differentiation 50 like by using tissue‐specific or regulatable promoters or by codelivering factors that restrain hypertrophy (basic fibroblast growth factor—FGF‐2, SOX transcription factors, anti‐Cbfa‐1 siRNA) 14, 15, 29, 48, 51, 52. It will be also important to determine the cell subpopulations of the aspirates involved in the chondroregenerative processes as well as the influence of environmental factors and molecular pathways on peripheral blood‐mediated cartilage repair. Altogether, the present results show the value of modifying such biological material to generate future, clinical treatment options for cartilage regenerative medicine.

## Conclusion

The present study describes the concept of applying rAAV vectors to achieve long‐term and high levels of transgene expression in human peripheral blood aspirates. More specifically, overexpression of the candidate TGF‐β factor via rAAV gene transfer was capable of enhancing the chondrogenic differentiation processes in the aspirates, providing a new, effective tool to treat cartilage lesions by transplantation of such genetically modified material upon controlled regulation of gene expression.

## Acknowledgments

We thank R.J. Samulski (The Gene Therapy Center, University of North Carolina, Chapel Hill, NC), X. Xiao (The Gene Therapy Center, University of Pittsburgh, Pittsburgh, PA), and E.F. Terwilliger (Division of Experimental Medicine, Harvard Institutes of Medicine and Beth Israel Deaconess Medical Center, Boston, MA) for providing genomic AAV‐2 plasmid clones and the 293 cell line. This work was funded by grants from the German Osteoarthritis Foundation (Deutsche Arthrose‐Hilfe e.V.).

## Author Contributions

J.F.: design, collection and/or assembly of data, data analysis and interpretation, manuscript writing, final approval of manuscript; P.O.: provision of study materials or patients, data analysis and interpretation, final approval of manuscript; J.K.V., A.R.‐R., and G.S.: collection and/or assembly of data, data analysis and interpretation, final approval of manuscript; D.K. and H.M.: data analysis and interpretation, final approval of manuscript; M.C.: conception and design, financial support, provision of study materials or patients, data analysis and interpretation, manuscript writing, final approval of manuscript.

## Disclosure of Potential Conflicts of Interest

The authors indicated no potential conflicts of interest.
